# Unexpected earthworm effects on forest understory plants

**DOI:** 10.1186/1472-6785-13-48

**Published:** 2013-12-05

**Authors:** Andrea Dávalos, Victoria Nuzzo, Jordan Stark, Bernd Blossey

**Affiliations:** 1 Department of Natural Resources, Cornell University, Ithaca, New York 14853, USA; 2 Natural Area Consultants, 1 West Hill School Road, Richford, New York 13835, USA; 3 TST New Visions in Life Sciences, Cornell University, Ithaca, New York 14853, USA; 4 Current address: Skidmore College, Saratoga Springs, New York 12866, USA

**Keywords:** *Lumbricus terrestris*, Forest understory, Plant community, Earthworm invasion, Mesocosm, Sedge

## Abstract

**Background:**

Introduced earthworms are widespread in forests of North America creating significant negative impacts on forest understory communities. However, much of the reported evidence for negative earthworm effects comes from field investigations either comparing invaded and non-invaded forests or across invasion fronts. While important, such work is rarely able to capture the true effect of earthworms on individual plant species because most forests in North America simultaneously face multiple stressors which may confound earthworm impacts.

We used a mesocosm experiment to isolate effects of the anecic introduced earthworm, *Lumbricus terrestris* L. on seedlings of 14 native plant species representing different life form groups (perennial herb, graminoid, and tree).

**Results:**

Earthworm presence did not affect survival, fertility or biomass of any of the seedling plant species tested over a 17-week period. However, *L. terrestris* presence significantly decreased growth of two sedges (*Carex retroflexa* Muhl. ex Willd. and *Carex radiata* (Wahlenb.) Small) by decreasing the number of culms.

**Conclusions:**

Our mesocosm results with seedlings contrast with field reports indicating extensive and significant negative effects of introduced earthworms on many mature native forbs, and positive effects on sedges. We suggest that earthworm impacts are context- and age-specific and that generalizations about their impacts are potentially misleading without considering and manipulating other associated factors.

## Background

The spread of non-native earthworms into previously earthworm-free temperate hardwood forests in North America can have significant ecosystem effects [1,2]. Earthworms can dramatically modify soil structure through reduction of the leaf litter layer and the top soil floor, redistribution of organic material, and changes in soil compaction and water flow, leading to changes in nutrient cycling, fine root distribution, microbial activity and reductions in litter-dependent biota [1-7]. Furthermore, earthworms can affect plant community assembly, plant survival and germination, both in Europe and North America, suggesting earthworms are important ecosystem engineers [3,8-12].

Observational evidence often indicates a correlation between earthworm presence and abundance of different plant species [3,13]. However, growth, survival, recruitment and demography of plant species interact with earthworms in multiple and complex ways, suggesting idiosyncratic species-specific relationships that can be highly dependent on habitat characteristics [8,14,15] and the influence of other associated stressors. For example, a study conducted in an aspen forest in the Rocky Mountains indicated that individual plant species presence, but not total plant biomass and cover, correlated with *Lumbricus terrestris* L. abundance, and that responses varied by plant species: *Vicia americana* Muhl. ex Willd. cover was negatively correlated, while *Viola canadensis* L. cover was positively correlated with *L. terrestris* abundance [13].

Several mechanisms have been proposed to explain patterns observed in the field. Plant species that may benefit from earthworm presence include species that expand vegetatively, produce small seeds, have high chemical protection, or do not form mycorrhizal associations [3]. Further, taxonomic and ecological characteristics of earthworms and plant species may also affect the outcome of their interactions [8,14,16]. Notably, life form and developmental stage may affect the importance of these different mechanisms. For example, seeds may be affected by direct consumption, while seedlings may be affected by desiccation of fine roots after leaf litter disappearance [9]. In addition, earthworm relative abundance and their associated impacts are also affected by land use history and grazing regimes [17], facilitation between earthworms and non-native plant species [7], and alterations of predator–prey interactions [6,18]. Furthermore, earthworms and abundant white-tailed deer (*Odocoileus virginiana* Zimmermann) in North America may interact and affect plants in multiple yet poorly understood ways. For example, earthworms benefit from high deer abundance by utilizing fecal pellets as a food resource [19], and earthworm invasion followed by the demise of vulnerable plants may also increase deer feeding pressure on remaining plants further reducing forest plant diversity [1].

While field studies have provided evidence for earthworm effects and generated plausible mechanisms for their impacts, a multitude of potentially confounding factors make it difficult to gauge the true extent of the earthworm effect. Predictions about detrimental or beneficial effects of earthworm invasion on different plant species appear difficult to make, and experimental studies are needed to identify and quantify the contribution of different mechanisms to the patterns reported from the field. While different venues clearly have effects on the outcome of ecological experiments and may even generate contradictory evidence [20,21], only through rigorous examination of multiple stressors and potential mechanisms in the same experimental framework can we advance our ecological understanding and gain an improved ability to manage ecological stressors. Here we evaluate, through a greenhouse mesocosm experiment, the effect of *L. terrestris* on seedlings of species typical of forest plant communities of central New York, USA. Our focal earthworm species *L. terrestris* was introduced by early European settlers and the species is now widely distributed across temperate North America. Impacts on plant communities by *L. terrestris* are believed to be mainly indirect, through changes in leaf litter abundance, humus and soil characteristics. For example removal of established humus and litter layers in forests that were previously earthworm-free, and subsequent mixing of topsoil, may lead to uprooting, desiccation and increased plant mortality [22]. However, *L. terrestris* can also produce significant direct effects via selective consumption of seeds and seedlings in both cotyledon and radical stages, especially of legumes [9]. In fact, seedling vulnerability to earthworm activities may be an Achilles heel for many native plant species. If the collapse of the leaf litter and humus layers is associated with widespread mortality of individuals, recruitment from the seed bank or dispersal by propagules from outside could rescue remaining populations from extinction or genetic bottlenecks. Such rescue effects, through natural processes or assisted restoration, will only be successful if seedlings are able to survive and grow after the initial earthworm invasion has changed local abiotic and biotic conditions.

Our goal was to evaluate whether seedlings of native forest understory plant species are able to survive and grow after initial earthworm impacts have occurred – i.e., in areas with no humus layer, but with annual leaf-litter inputs. We used large experimental units (tree pots) to allow proper burrowing of *L. terrestris* and to provide enough space to grow multiple plant species as earthworm and plants would naturally encounter in the field. We focused on the seedling stage and therefore planted seedlings rather than seed, to avoid confounding different mechanisms (such as direct consumption) operating at different developmental stages. While direct consumption or burial of seed is clearly an important mechanism, seed predation is unlikely to remove 100% of propagules, so we were interested in the performance of surviving individuals.

We selected 14 plant species that occur in deciduous forests of central New York (Table 1); all are common with the exception of the state-endangered *Aristolochia serpentaria* L. and *Carex retroflexa* Muhl. ex Willd. We included five species predicted to be favored by earthworm presence (two grasses, two sedges and an ephemeral forb), two species predicted to be harmed by earthworm presence (two trees), and seven species for which we had no *a priori* predictions (Table 1) since these species had not previously been studied for their response to earthworm invasions. In our investigations we were guided by the following hypotheses: (1) plant responses will be species-specific, therefore effects on survival, flowering and plant growth will depend on species identity and life form; (2) survival, flowering and growth of grasses and sedges will increase in the presence of *L. terrestris*; and (3) survival, flowering and growth of forbs and woody seedlings will decrease in the presence of *L. terrestris*.

**Table 1:** **Family, life form, age, predicted response to *L. terrestris *** **presence and MANOVA results on plant size measures according to earthworm treatment of 14 native plant species planted in experimental mesocosms**

**Species**	**Abbreviation**	**Family**	**Life form**	**Seedling age**	**Predicted response**	**MANOVA response**^**a**^	**df**	**F**^**c**^	**P**
*Agrimonia gryposepala* Wallr	Agr	Rosaceae	Forb	7 m	----	Belowground, aboveground biomass, height	3,32	0.29	0.83
*Agrostis hyemalis* (Walter) BSP	Ahy	Graminae	Grass	2 m	Positive^1^				
*Allium tricoccum* Aiton	Atr	Alliaceae	Forb	9 m	Positive^2,3^				
*Aristolochia serpentaria* L.	Ase	Aristolochiaceae	Forb	1 m	----	Belowground, aboveground biomass, height, # of leaves^b^	4,28	0.25	0.90
*Carex radiata* (Wahlenb.) Small.	Cra	Cyperaceae	Sedge	1 m	Positive^3,4^				
*Carex retroflexa* Muhl. ex Willd.	Cre	Cyperaceae	Sedge	1 m	Positive^3,4^				
*Elymus hystrix* L.	Ehy	Graminae	Grass	7 m	Positive^1^				
*Eurybia divaricata* L.	Edi	Asteraceae	Forb	7 m	----				
*Phryma leptostachya* L.	Ple	Phyrmaceae	Forb	7 m	----	Belowground, aboveground biomass, height	3,25	0.72	0.54
*Quercus prinus* L.	Qpr	Fagaceae	Tree	3 m	Negative^4,5^	Belowground, aboveground biomass, height, # of leaves^b^	4,31	0.68	0.57
*Quercus rubra* L.	Qru	Fagaceae	Tree	1wk	Negative^4,5^	Belowground, aboveground biomass, height, # of leaves^b^	4,31	0.94	0.43
*Ranunculus recurvatus* Poir.	Rre	Ranunculaceae	Forb	7 m	----				
*Tiarella cordifolia* L.	Tco	Saxifragaceae	Forb	7 m	----				
*Viburnum lantanoides* Michx*.*	Vla	Adoxaceae	Tree	7 m	----	Belowground, aboveground biomass, height	3,31	1.45	0.25

*Abbreviations:m* month, *wk* week. ^a^MANOVA was run only for plant species for which we measured multiple size variables. ^b^Variable was log-transformed. ^c^F-values were approximated from Pillai–Bartlett statistic; ^1^[14]; ^2^[40]; ^3^[3]; ^4^[41]; ^5^[48].

## Methods

We conducted our experiment from 11 January to 9 June 2011 in a temperature controlled greenhouse at Cornell University (constant 19°C). Greenhouse temperature was within the thermal tolerance of *L. terrestris*[23] and reflected average temperature during the growing season in Ithaca, NY [24]. We did not manipulate the photoperiod, which increased throughout the duration of our experiment. We established 36 mesocosms consisting of 140 L plastic tree-pots (diameter 60 cm, 50 cm tall), in which we placed a fine mesh bag to prevent earthworm escape. We placed a 5 cm layer of sand at the bottom of each pot and added moist Cornell Potting Mix (Cornell University, Ithaca NY) until pots were filled to the 40 cm mark. Cornell Potting Mix is a soilless mixture composed of peat moss (0.9 – 0.1 m^3^ / 0.92 m^3^), vermiculite (0.06 – 0.17 m^3^/ 0.92 m^3^), dolomitic limestone (9 kg / 0.92 m^3^) and fertilizer (2.72 kg / 0.92 m^3^, N:P:K = 11:5:11) [25]. Due to the number and large size of mesocosms we elected to use an artificial potting medium rather than autoclave (which eliminates both soil structure and soil microbes) or hand sort field-collected soil to remove soil-dwelling organisms. Micro and macro soil organisms may affect earthworm or plant performance and their absence from the potting medium enabled us to isolate the direct earthworm effect that we sought to capture. Guidelines for testing chemical toxicity on earthworms recommend the use of a standardized artificial medium [26] and studies using soil-free potting medium report normal earthworm survival and activity [27,28]. Chemical analyses of Cornell Potting Mix indicated that the potting mix composition was similar to artificial media (JRPeters Laboratory, Allentown PA), and nutrient comparisons between the potting mix and soil samples taken at 12 forest sites with varying earthworm density at West Point NY (unpublished data) indicated no significant differences in pH (5.98 vs. 4.98 ± 0.56 mean ± SE) or in primary soil nutrients: P (15.3 ppm in potting mix vs. 11.52 ± 5.08 in forest samples), K (110 ppm vs. 172.09 ± 37.70) and Ca (67 ppm vs. 93.19 ± 14.68).

In each pot we created a grid of 14 evenly spaced planting locations (about 6 cm apart), and on 19 January we transplanted one seedling of each target plant species into each mesocosm following a pre-determined randomization pattern. We replaced seedlings that died within the first two weeks after transplanting. We watered seedlings every 2–4 d for the first two weeks, and then every 4–7 d for the remainder of the experiment.

We selected 14 native plant species representing different life forms, genera, and families (Table 1). We grew all seedlings from seed during the previous year(s), or from fall-collected seed (*Quercus* spp.). Within each species, seedlings were of the same age and size in both treatments, but among species seedlings varied in age from 1 week to 9 months (Table 1). We propagated the majority of species in individual seedling cells (3.8 × 3.8 × 6 cm) in summer 2010, held overwinter in a cold-room or cold-frame, and transferred them to a warm environment on 12 January 2011. Seedlings were emerging from the soil at the time of experimental plantings.

After seedling establishment (18 d) we added 70 g of leaf litter to each mesocosm (40 g *Acer saccharum* Marsh, 30 g *Fraxinus americana* L. collected the previous fall and dried at 80°C for 72 h). To allow for faster decomposition and easier access by earthworms we shredded dried leaves into 1–2 cm diameter pieces and moistened them before adding litter.

We hand collected sexually mature *L. terrestris* in November 2010 in Lansing, NY and kept them in 20 L plastic bins covered with mesh and filled with a shallow layer of potting soil and leaves in a refrigerator at 5°C until they were used in our experiment. On 10 February we randomly assigned mesocosms to one of two earthworm treatments (*L. terrestris* present or absent, N = 18 per treatment) and added six *L. terrestris* per pot (mean fresh biomass 16.83 to 19.53 g). This earthworm density is comparable to the average density of adult *L. terrestris* found over three years of monitoring (2008–2010) at West Point, NY (4.88 individuals per 0.25 m^2^) and to reported densities at other sites in central New York [5]. After introduction of earthworms, we sealed each mesh bag at the top to prevent earthworm escape. We kept mesh bags upright by placing three 90 cm bamboo support stakes into each tree pot.

On 31 March and 13 April we recorded survival of all plant species and counted the number of *C. retroflexa* and *C. radiata* culms. After 17 weeks (6–10 June), we terminated the experiment. We collected all remaining leaf litter from each pot and then carefully removed each plant including all belowground structures; we were careful to avoid breaking fine roots or damaging earthworms. We recorded survival for all species; measured height for *A. gryposepala, A. serpentaria, P. leptostachya, Q. prinus, Q. rubra* and *V. lantanoides*; number of culms for *C. radiata* and *C. retroflexa*; number of leaves for *A. serpentaria, Q. prinus* and *Q. rubra;* and presence/absence of flowers for *A. gryposepala, A. hyemalis, E. divaricata, C. radiata, C. retroflexa, E. hystrix, P. leptostachya, and R. recurvatus.* We separated roots from stems and leaves and carefully washed each plant to remove any soil particles. We kept above and belowground structures separate and determined their biomass and the biomass of the remaining litter after drying material at 80°C for 72 h; due to senescence we could record only belowground biomass for *A. tricoccum*. After removing all plant material, we extracted remaining earthworms by pouring 3.79 L of mustard solution at 15 g L^-1^on each pot (Frontier Natural Products Co-op, Norway, IA).

### Statistical analyses

We assessed differences in leaf litter biomass between earthworm treatments with a one-way ANOVA. We fitted a Generalized Linear Model (GLM) with binomial errors to evaluate the effect of earthworm treatment, plant life form and plant species (nested within life form) on plant survival. We evaluated earthworm effects on the probability of flowering of *A. gryposepala* and *A. hyemalis* with a second GLM. We only evaluated these two species because flowering of the remaining species was too low to adequately fit the model. In order to account for differences in plant productivity between pots, we included total plant biomass per pot as a covariate in both sets of models.

We ran 2-way ANOVAS to evaluate the effect of earthworm treatment and plant life form (forb, grass, sedge or tree) on aboveground, belowground or total biomass per pot (independent models for each response variable) and on the ratio of above to below ground biomass (*A. tricoccum* was excluded from this analysis because it senesced before harvest). We then analyzed the effect of earthworm treatment on plant size with separate Multivariate Analysis of Variance, (MANOVA) for plant species for which we measured multiple size variables. Response variables varied between species (Table 1). We used a Mixed Linear Model to analyze the effect of earthworm treatment and time on number of culms of *C. retroflexa* and *C. radiata* (separate models for each species). We included pot identification as a random factor to account for repeated measures nature of the data. Number of culms was log transformed to comply with model assumptions.

We examined and confirmed that test assumptions were met for all cases. We conducted all tests in R 2.14 [29], and we fitted Mixed Models with the lme4 package [30].

## Results

**Figure 1:**
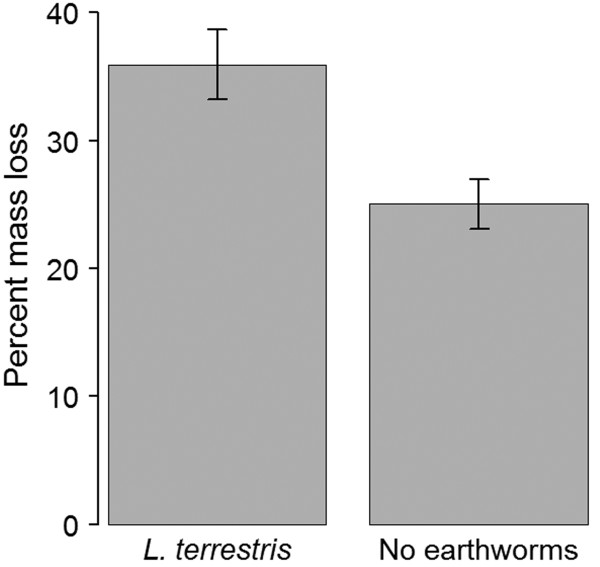
**Leaf litter mass loss (expressed as percent of initial total leaf dry mass) according to earthworm addition treatment. Data are means + 2SE, N** = **18 mesocosms.**

We observed middens and castings in all treatment pots throughout the experiment indicating active earthworms. As expected, earthworm activity significantly accelerated litter decomposition, with 15% lower litter biomass in treatment pots compared to control pots (F_1,34_ = 10.75, P = 0.002; Figure 1). At the end of the experimental period, we recovered 1–4 individual *L. terrestris* from 11 of the 18 stocked mesocosms and found no earthworms in the control mesocosms. Thus, although earthworm recovery rate was low, earthworms were present and active in all treatment pots and their effect was clearly evident in the observed litter reductions, allowing us to assess earthworm effect on plant performance.

Survival of our transplanted individuals was very high (95.6 ± 0.01%) with significantly lower survival for *A. tricoccum* (83%) and *Tiarella cordifolia* (88%) than for the remaining plant species (97% to 100%; z = −3.05, P = 0.002). Grass (98%; z = 2.30, P = 0.03) and tree survival (98%; z = 2.72, P = 0.006) was higher than forb and sedge survival (94%). Survival was not affected by total plant biomass per plot, earthworm treatment or the interaction between plant life form and treatment (P > 0.05 for all cases).

The probability of flowering was significantly higher for *A. gryposepala* than for *A. hyemalis* (z = 3.08, P = 0.002; 86% vs. 31% of plants flowered, respectively), but was not affected by earthworm treatment, the interaction between plant species and earthworm treatment or total biomass per pot (P > 0.05 for all cases). We recorded flowering for *P. leptostachya* (17%)*, E. hystrix* (14%), *R. recurvatus* (6%), *C. retroflexa* (6%) and *E. divaricata* (3%), but due to the low number of flowering plants in these species we did not evaluate a potential *L. terrestris* effect.

**Figure 2:**
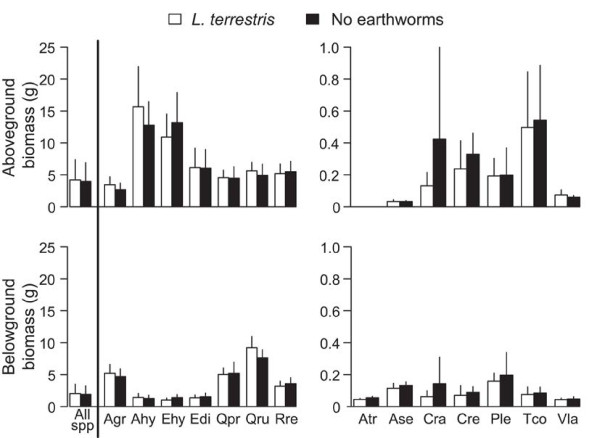
**Aboveground (top panels) and belowground (bottom) biomass (g) of plant species grown in presence/absence of *L. terrestris*.** For clarity, plant species with high (> 1 g) and low (< 1 g) biomass are shown separately in the left and right panels, respectively. Plant species are ordered alphabetically and their names are a combination of the first letter of the genus followed by the first two letters of the species epithet. For a complete list please review Table 1. Data are means + 2SE, N = 18 mesocosms.

**Figure 3:**
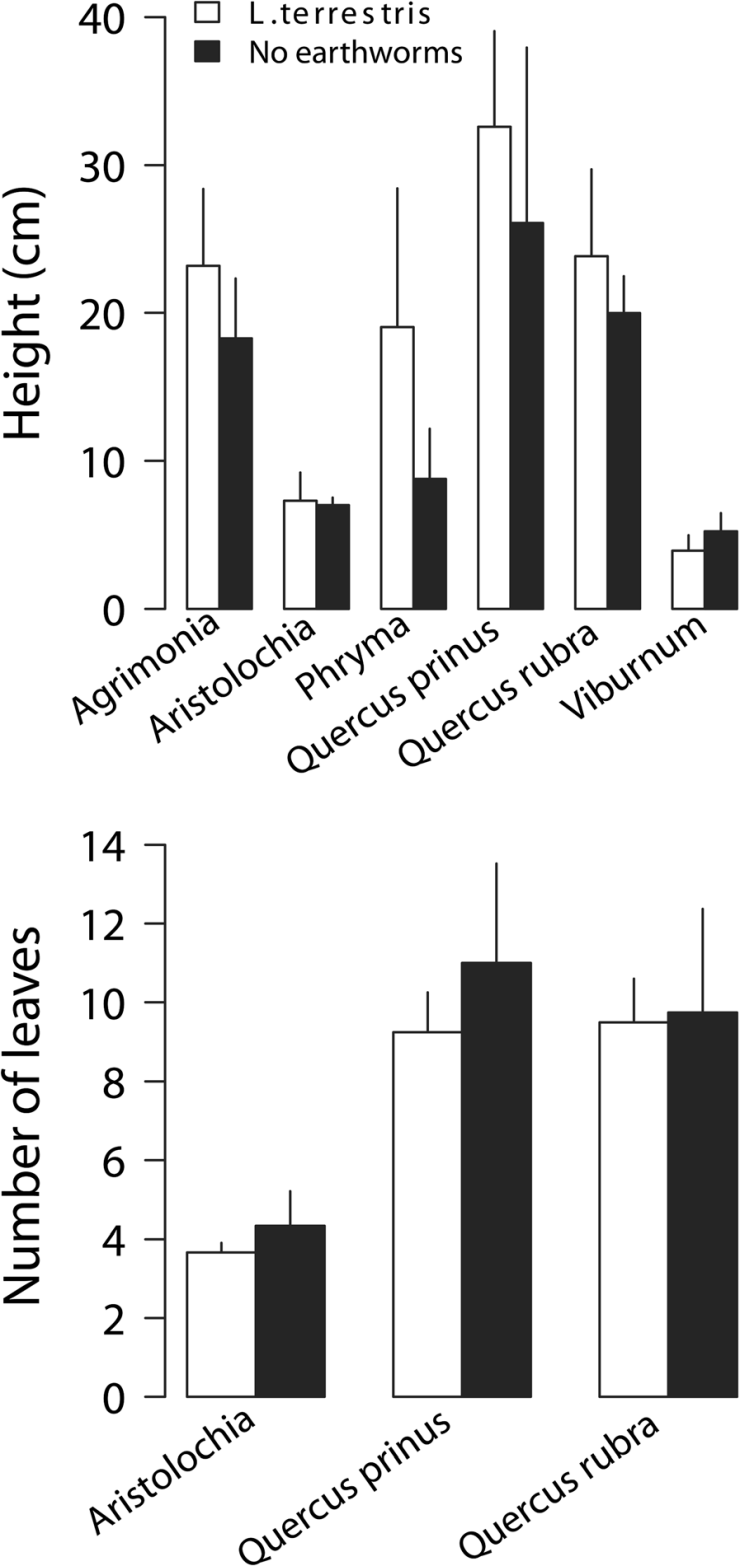
**Plant height (top) and number of leaves (bottom) of plant species grown in presence/absence of *L. terrestris*.** We collected additional measurements for a subset of species only. Please refer to methods sections for details. Data are means + 2SE, N = 18 mesocosms.

Aboveground, belowground and total biomass per pot, and the ratio of above to belowground biomass were not affected by earthworm treatment but they significantly differed among life forms, with grasses attaining the highest biomass (Table 2; Figure 2). We did not find a significant interaction between earthworm treatment and plant life form. Independent analyses of each species indicated no difference in plant size in the presence or absence of earthworms for any plant species (MANOVA results, Table 1; Figure 3).

**Table 2:** ANOVA results for the effects of earthworm addition and life form on plant aboveground, belowground and total dry biomass (g), and on the ratio of above to belowground biomass

**Response**	**df**	**Above**		**Below**		**Total**		**Ratio**	
		**F**	**P**	**F**	**P**	**F**	**P**	**F**	**P**
Earthworm addition	1, 135	0.02	0.87	0.79	0.38	0.10	0.75	2.61	0.11
Life form	3, 135	61.43	<0.001	156.99	<0.001	56.64	<0.001	54.91	<0.001
Interaction	3, 135	1.44	0.23	0.12	0.95	1.29	0.28	1.29	0.28

**Figure 4:**
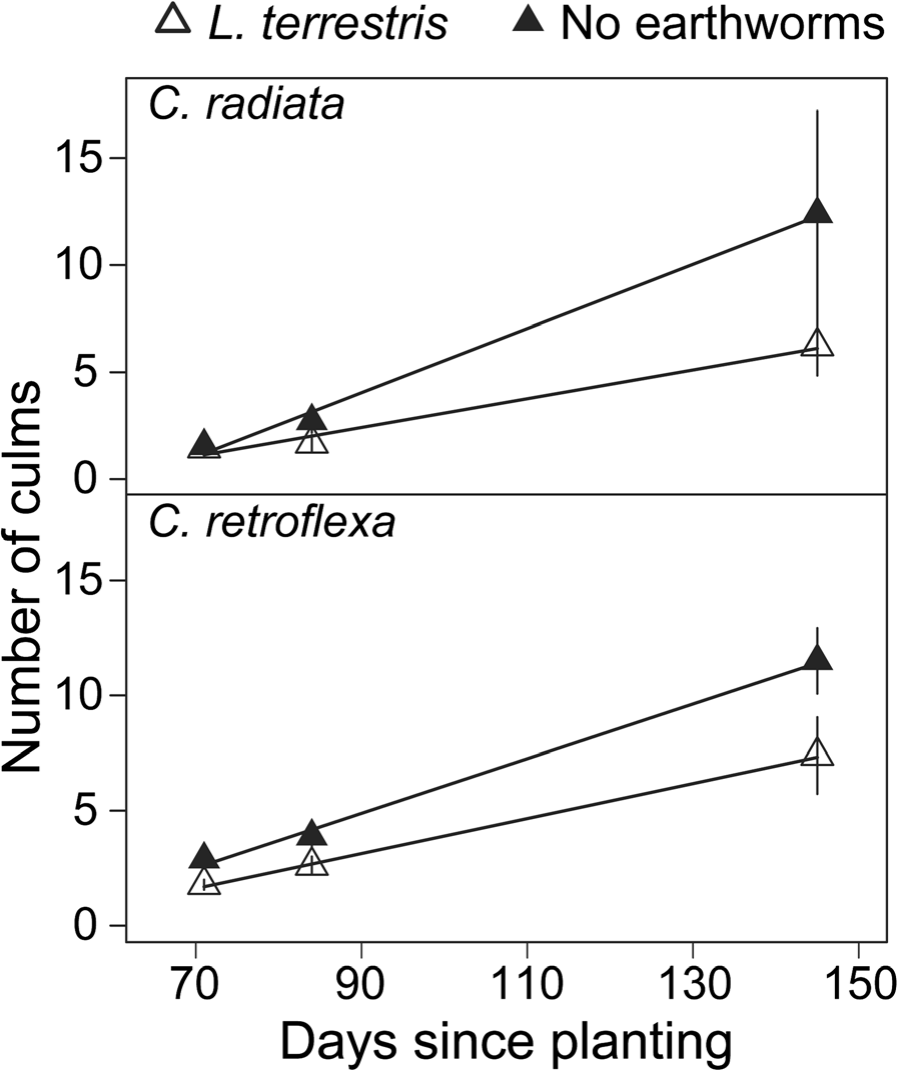
**Mean number of *Carex radiata *(top) and *C. retroflexa *(bottom) culms grown in presence/absence of *L. terrestris*.** Error bars are ± 2SE, N = 18 mesocosms. Lines depict model predictions. Earthworm treatments were imposed 22 d after transplanting of the seedlings.

As expected the number of culms of the sedge species, *C. radiata* and *C. retroflexa,* increased significantly over the course of the experiment. However, earthworms significantly slowed the rate of this clonal expansion (significant time x earthworm addition interaction, Table 3). By the end of the experiment, both species had significantly fewer culms in the presence of earthworms: *C. radiata* averaged a 50% reduction (Figure 4 top), and *C. retroflexa* averaged a 36% reduction (Figure 4 bottom).

**Table 3:** **Mixed linear model results for the effects of earthworm addition and time on the number of culms of *Carex radiata *** **and *Carex retroflexa***

**Response**	***Carex radiata***			***Carex retroflexa***		
	**Estimate**	**SE**	**t value**	**Estimate**	**SE**	**t value**
Earthworm addition	−0.08	0.28	−0.30	0.03	0.025	0.1
Time	0.01	0.0001	8.25***	0.01	0.001	8.85***
Interaction	−0.004	0.002	2.11*	0.003	0.001	2.1*

*** P < 0.0001, *P < 0.05.

## Discussion

Contrary to our expectations, our results do not indicate widespread significant negative effects of *L. terrestris* on forest plant seedlings in mesocosms. We did not detect a negative impact on survival, growth or reproductive effort for 12 of the 14 species in our experiment, but did detect a negative effect on two species thought to benefit from earthworm presence. Our experimental design and results do not address any initial effect of earthworm invasions on plant survival [3], as earthworms destroy humus and litter layers through their feeding activities (which also happened in our experiment), nor on germination from the seed bank after initial invasion. Our results do, however, indicate that this negative effect may be temporary and that most plants may be able to coexist with *L. terrestris* after initial impacts have occurred.

Further, our results indicate that for our experimental plant species, seedlings that have developed several leaves are not overly vulnerable to earthworm activity. The often reported lack of plant recruitment after earthworm invasion may be a function of reduced rescue of populations from a depauperate seed bank [31-33], detrimental effects of earthworms on seeds via direct consumption or burial [34] or maternal effects [35], and by direct consumption of cotyledons [9] of early recruiting seedlings. Clearly more work in different venues and with manipulations of other stressors is required to better understand the ultimate cause of the reported depauperate plant communities after initial earthworm invasions [3].

We used leaf litter disappearance as a cumulative indicator of earthworm activity throughout the course of the study to account for the low earthworm recovery rate at the end of the experiment. The low earthworm recovery rate might be explained, at least partially, by the inefficiency of the mustard extraction method. Assessments of the efficiency of this method indicate that the mustard solution only extracts a portion of earthworms, favors certain species and is overall less efficient than hand sorting [36,37]. Despite the low earthworm recovery rate, leaf litter disappearance was significantly higher in earthworm mesocosms, indicating that earthworms were active throughout the experiment. After 110 d of exposure, our earthworm mesocosms had an average of 65% leaf litter remaining (Figure 1), a higher disappearance rate compared to that of leaf litter placed in field boxes at earthworm-invaded sites (earthworm density = 79.6 ± 10.2 earthworms per m^2^) in New York State after 190 d of exposure [5]. Leaf litter disappearance is associated with earthworm invasion and often used as an indicator of their impacts [5,38]. We obtained similar results regardless of whether we used initial earthworm treatments, remaining leaf litter biomass at the end of the experiment or final earthworm presence as predictor variables and we are therefore confident that our anticipated treatments were realized.

Our findings also support previous results indicating that earthworm impacts are context and species-specific [39-41]. From the 14 plant species tested, only the two sedges were negatively affected by *L. terrestris*. While sedges in general are thought to benefit from earthworm presence [3,42], our results indicate a negative effect of *L. terrestris* on *Carex radiata* and *C. retroflexa* growth rate, as both species produced fewer culms in the presence of this earthworm. The positive association between sedge cover and earthworm abundance observed in field studies is highly dependent on earthworm and plant species. For example, cover of *Carex pensylvanica* Lam. in northern Wisconsin is positively associated with biomass of earthworm species in the genera *Allolobophora* and *Aporrectodea*, but not with species in the genera *Lumbricus, Octolasion or Dendrobaena*[40]. Further, while the common sedge *C. pensylvanica* can form monotypic stands when earthworms are present [3,41], the endangered sedge, *Carex deweyana* Schwein., is negatively associated with earthworm abundance [40].

Earthworms can disrupt mycorrhizal associations [43] and therefore it has been hypothesized that non-mycorrhizal species, such as *Carex pensylvanica*, may benefit from their presence [3]. Although Cyperaceae is commonly considered a non-mycorrhizal family, several *Carex* species can form and benefit from mycorrhizal associations [44,45]. Variable responses among *Carex* species to earthworm invasion may be a result of some species dependence on mycotrophy, but confirmation of this and other potential, but currently elusive, mechanisms of variable earthworm impacts require careful experimentation. While we cannot identify the specific mechanism, we conclude that earthworm impacts occurred, potentially through changing composition and effects of soil microbial communities.

We did not find any indication of a differential effect of *L. terrestris* on distinct plant life forms. Contrary to our expectations productivity of grasses and sedges was not higher in earthworm mesocosms. In a previous microcosm study grass productivity increased with short- and long-term presence of *L. terrestris*, likely due to increased N from worm activity [14]. In our study, N was readily available, allowing us to separate the effect of earthworm presence and activity from the effect of nutrient addition. The effects of the assembled plant community via resource availability and plant-plant interactions can substantially modify effects of earthworms. For example, growth of *Hordeum vulgare* L. increased in presence of earthworms when the plant was grown in monoculture but not in polyculture, suggesting that the ability of grasses to benefit from increased nutrients in earthworm presence is reduced by the presence of forbs [46].

We focused on impacts of a single anecic earthworm species. Other researchers have shown that earthworm species from different ecological groups (endogeic, epigeic or anecic) produce singular impacts, which are usually more severe when several earthworm species are present [10] and impacts may even vary within the same ecological group [47]. For example, in our mesocosms, both oak species were unaffected by *L. terrestris* presence. In a field experiment, however, transplanted red oak seedlings (*Quercus rubra*) had reduced growth in sites with abundant earthworms than in sites with no earthworms [48]. In these sites up to 10 earthworm species, but not *L. terrestris*, were identified, indicating that species identity, ecological group and species richness play an important role on the magnitude of the impacts.

Despite the benefits of mesocosm studies to isolate mechanisms, they do present shortcomings and require field verification of effects in additional studies. Our mesocosms lacked soil structure and plants were kept well watered, preventing at least two main mechanisms by which *L. terrestris* is believed to produce negative impacts: mixing of soil layers and desiccation. These mechanisms can be explored using field soil in small microcosms, although *L. terrestris* movement is compromised in small containers. For large mesocosms such as our 140 L units, it is logistically challenging to extract intact soil cores while maintaining soil structure, followed by autoclaving or hand sorting to eliminate earthworms and other soil biota. Despite the successful use of soilless substrates in toxicology studies [27,28], it is possible that the substrate might have affected earthworm behavior. However, there is no evidence to evaluate this claim, raising the need for a formal assessment of the effects of artificial and natural substrates on earthworm activity.

If many forests invaded by earthworms now lack a previously existing herbaceous layer or are dominated by sedges, it appears necessary to go beyond earthworms as sole causal agents. Land use history and interactions with other plant stressors, such as introduced plants, overgrazing by ever expanding white-tailed deer herds and nutrient deposition are likely to influence the observed patterns. Without further experimental manipulations of these various stressors in the field and in experimental venues, our ability to predict the strength of earthworm impacts compared to the importance of other stressors remains limited. However, most importantly, our results indicate that in forests invaded by *L. terrestris*, which lack a humus layer and are only covered by annual leaf litter inputs, many native plant species should be able to survive and reproduce once other conditions (such as deer overgrazing) are remedied. Thus, restoration of a diverse forest floor community is possible if seedlings emerge from the seed bank, or are planted through active restoration.

## Conclusions

Our results indicate no effects of earthworms on seedling survival, biomass or fertility of 12 of the 14 plant species tested when grown in experimental mesocosms. Contrary to previously reported results, we found a negative effect of *L. terrestris* presence on growth rate of two sedge species: *C. retroflexa* and *C. radiata.* Results suggest that earthworm effects are context specific and that field experimentation is necessary to understand earthworm individual and combined effects on plant communities. Our results also indicate that it is feasible to restore understory flora in forests colonized by non-native earthworms.

## Competing interests

All authors declare no competing interests.

## Authors’ contributions

All authors contributed to study design and interpretation of results. VN grew all plants and coordinated the study, JS did the majority of data collection and AD analyzed the data and wrote the majority of the paper with contributions from all co-authors. All authors have read and approved the final manuscript.
